# Augmentative and alternative communication in children with Down’s syndrome: a systematic review

**DOI:** 10.1186/s12887-018-1144-5

**Published:** 2018-05-11

**Authors:** Renata Thaís de Almeida Barbosa, Acary Souza Bulle de Oliveira, Jennifer Yohanna Ferreira de Lima Antão, Tânia Brusque Crocetta, Regiani Guarnieri, Thaiany Pedrozo Campos Antunes, Claudia Arab, Thaís Massetti, Italla Maria Pinheiro Bezerra, Carlos Bandeira de Mello Monteiro, Luiz Carlos de Abreu

**Affiliations:** 1 Laboratório de Delineamento de Estudos e Escrita Científica, Departamento de Saúde da Coletividade, Faculdade de Medicina do ABC (FMABC), Rua Fagundes Varela, 121. Vila Príncipe de Gales, 09060510 Santo André, SP Brasil; 2 Escola Paulista de Medicina, Disciplina de Neurologia Clínica. Setor de Doenças Neuromusculares, Universidade Federal de São Paulo (UNIFESP-EPM), Rua Botucatu, 740, Vila Mariana, 04023900 São Paulo, SP Brasil; 3 Escola Paulista de Medicina. Disciplina de Cardiologia, Departamento de Medicina, Universidade Federal de São Paulo (UNIFESP), Rua Napoleão de Barros, 715, Vila Clementino, 04023062 São Paulo, SP Brasil; 4 Programa de Pós-Graduação em Ciências da Reabilitação, Faculdade de Medicina, Universidade de São Paulo (USP), Rua Cipotânea, 51, Cidade Universitária, 05360000 São Paulo, SP Brasil; 5 Escola Superior de Ciências da Santa de Misericórdia de Vitória (EMESCAM), Av. N.S da Penha, 2190, Santa Luiza, 29045402 Vitória, ES Brasil; 6 Escola de Artes, Ciências e Humanidades, Universidade de São Paulo (EACH-USP), Rua Arlindo Béttio, 1000, Ermelino Matarazzo, 03828000 São Paulo, SP Brasil; 7 Departamento de Saúde Materno Infantil, Faculdade de Saúde Pública, Universidade de São Paulo (USP), Av. Dr. Arnaldo, 715, 01246904 São Paulo, SP Brasil

**Keywords:** Down’s syndrome, Children, Assistive technology, Augmentative and alternative communication

## Abstract

**Background:**

The use of technology to assist in the communication, socialization, language, and motor skills of children with Down’s syndrome (DS) is required. The aim of this study was to analyse research findings regarding the different instruments of ‘augmentative and alternative communication’ used in children with Down’s syndrome.

**Methods:**

This is a systematic review of published articles available on *PubMed*, *Web of Science*, *PsycInfo*, and *BVS* using the following descriptors: *assistive technology AND syndrome*, *assistive technology AND down syndrome*, *down syndrome AND augmentative and alternative communication*. Studies published in English were selected if they met the following inclusion criteria: (1) study of children with a diagnosis of DS, and (2) assistive technology and/or augmentative and alternative communication analysis in this population.

**Results:**

A total of 1087 articles were identified. Thirteen articles met the inclusion criteria. The instruments most used by the studies were speech-generating devices (SGDs) and the Picture Exchange Communication System (PECS).

**Conclusion:**

Twelve instruments that provided significant aid to the process of communication and socialization of children with DS were identified. These instruments increase the interaction between individuals among this population and their peers, contributing to their quality of life and self-esteem.

**Electronic supplementary material:**

The online version of this article (10.1186/s12887-018-1144-5) contains supplementary material, which is available to authorized users.

## Background

According to the World Health Organization (WHO), one in every 1100 children born worldwide harbours a chromosome 21 genetic abnormality. In the United States, 250,000 families are affected by Down’s syndrome (DS) [1] with a prevalence of one per 691 live births [2].

DS is a disorder caused by trisomy of human chromosome 21 (Hsa21) and presents various anomalies of the respiratory, cardiovascular, sensory (organs), gastrointestinal, haematological, immune, endocrine, musculoskeletal, renal, and genitourinary systems [3]. Furthermore, individuals with DS may have changes in body anatomy, such as different facial features with a rounded and flat face, an epicanthic fold, an oblique palpebral cleft, dysmotic ears, a flat and flattened nose, a short and wide neck, a small mouth with hypotonic tongue with tongue protrusion, brachycephaly, short stature, hands that may present clinodactyly or syndactyly, a single palmar fold, and small feet [3, 4]. With regard to development and cognitive aspects, this population can present difficulty in learning [5]. For example, the majority of children with DS exhibit a moderate degree of intellectual disability [4, 6], a low Intelligence Quotient (IQ), and low memory [7].

Considering the cognitive aspects, growth, and learning process associated with psychological, cultural, and environmental factors is especially important for children with DS because they need to be integrated into society and have autonomy and independence in their activities [8]. Given that DS is a common chromosomal alteration in humans and one of the leading causes of intellectual disability in the world population, it is extremely important to use tools that help in the development of communication to provide better socialization [9].

One possibility of supporting the communication process is through tasks that are fun and provide cognitive and motor stimuli, especially for those with communication deficits who often need to use complementary, additional or amplified communication systems to establish an interaction process [10]. Thus, one technology specifically designed to help individuals without functional speech or writing or with a gap between their communicative need and their actual ability to communicate (speak and/or write) is ‘augmentative and alternative communication’ (AAC) [11].

AAC includes aided communication modes that require additional materials or devices and is subdivided into high and low-technology AAC. Low-technology systems or devices encompass communication books or boards (non-powered), written words on paper, photographs, line drawings and pictograms. High-technology systems include voice output communication aids (VOCAs), which are known as ‘speech-generating devices’ in North America, and software on personal computers or laptops used as communication aid (providing recorded or synthesized voice or written output). Moreover, the concept includes technology that provides access to personal computers or laptops, enabling their use as communication aids [12].

According to Foreman and Crews [8], children with DS often present difficulties in ​​language and communication as well as visual and perceptual areas, thereby suggesting that they may potentially benefit from using AAC systems to improve language development, communication, and consequently the socialization process. In this context, AAC is a key area of research aimed at studying and developing mechanisms, tools, and methodologies to complement, supplement, or increase the potential for communication [10] and has been widely used with different disorders.

Some studies highlight the importance of the relevance of the intervention for the AAC. For example, Soto and Clarke [13] demonstrated the positive effects of the conversation-based intervention for improving the expressive vocabulary and grammatical skills of children with severe motor speech disorders and expressive language delay who use augmentative and alternative communication. Moreover, their study discusses clinical and theoretical implications of conversation-based interventions and identifies future research needs in the area*.* Finke et al. [14] studied school-age children with autism spectrum disorder (ASD) and identified benefits associated with the use of AAC with high-technology devices for multi-symbol messages. In a review with adults with post-stroke aphasia, Russo et al. [15] described a compensatory strategy to enhance communicative skills with AAC technology.

With regard to DS, several systemic reviews have been reported on observed reading skills [16], language and verbal short-term memory skills [17], and cognitive and behavioural functioning across the lifespan [18] as well as studies on reading comprehension in developmental disorders of language and communication [19]. However, no review studies have been found that observed the instruments used for AAC for DS.

Therefore, the aim of this study is to investigate the results presented in previous studies (i.e., clinical trials, case-control, cross-sectional, case reports, and case series) on AAC use in children with DS observing the different instruments used for communication. The presentation of existing knowledge about technological modernity and this new communication tool in DS can help in the organization of treatment programmes and benefits aimed at improving the communication and independence of this population.

## Methods

### Search strategy

This review was based on a systematic search conducted in August 2017 of published articles available on *PubMed* (http://www.ncbi.nlm.nih.gov/pubmed), *Web of Science* (https://webofknowledge.com/), *BVS* – Virtual Health Library (http://bvsalud.org/), and *PsycInfo* (http://www.apa.org/pubs/databases/psycinfo/index.aspx) using keywords obtained from the Health Sciences Descriptors (DeCS) of the Virtual Health Library. The searches were conducted thrice on each database (See Table 1). We used the descriptor ‘*syndrome*’ in all searches to ensure that all potential articles were obtained. The review was performed according to the Preferred Reporting Items for Systematic Reviews and Meta-Analyses (PRISMA) guidelines [20]. The use of checklists, e.g., PRISMA, improves the reporting quality of systematic reviews and provides substantial transparency in the article selection process [21, 22].

**Table 1:** Studies searches according to database, terms and quantity of returned studies

Database	Search terms	Articles returned, n
1st search in Web of Science	Topic: (assistive technology AND syndrome) AND (language:(“English”) AND type:(“article”)	45
2nd search in Web of Science	Topic: (assistive technology AND down syndrome) AND (language:(“English”) AND type:(“article”)	4
3rd search in Web of Science	Topic: (down syndrome AND augmentative and alternative communication) AND (language:(“English”) AND type:(“article”)	17
1st search in PubMed	(Search details): (assistive technology [All Fields] AND syndrome [All Fields])	218
2nd search in PubMed	(Search details): (assistive technology [All Fields] AND down syndrome [All Fields])	25
3rd search in PubMed	(Search details): (down syndrome [All Fields] AND augmentative and alternative communication [All Fields])	20
1st search in PsycInfo	Any Field: augmentative AND Any Field: alternative communication AND Any Field: “down syndrome”	0
2nd search in PsycInfo	Any Field: assistive technology AND Any Field: “down syndrome”	0
3rd search in PsycInfo	Any Field: assistive technology AND Any Field: syndrome	56
1st search in BVS	All fields: assistive technology AND syndrome	655
2nd search in BVS	All fields: assistive technology AND down syndrome	14
3rd search in BVS	All fields: down syndrome AND augmentative AND alternative communication	33

**Fig. 1:**
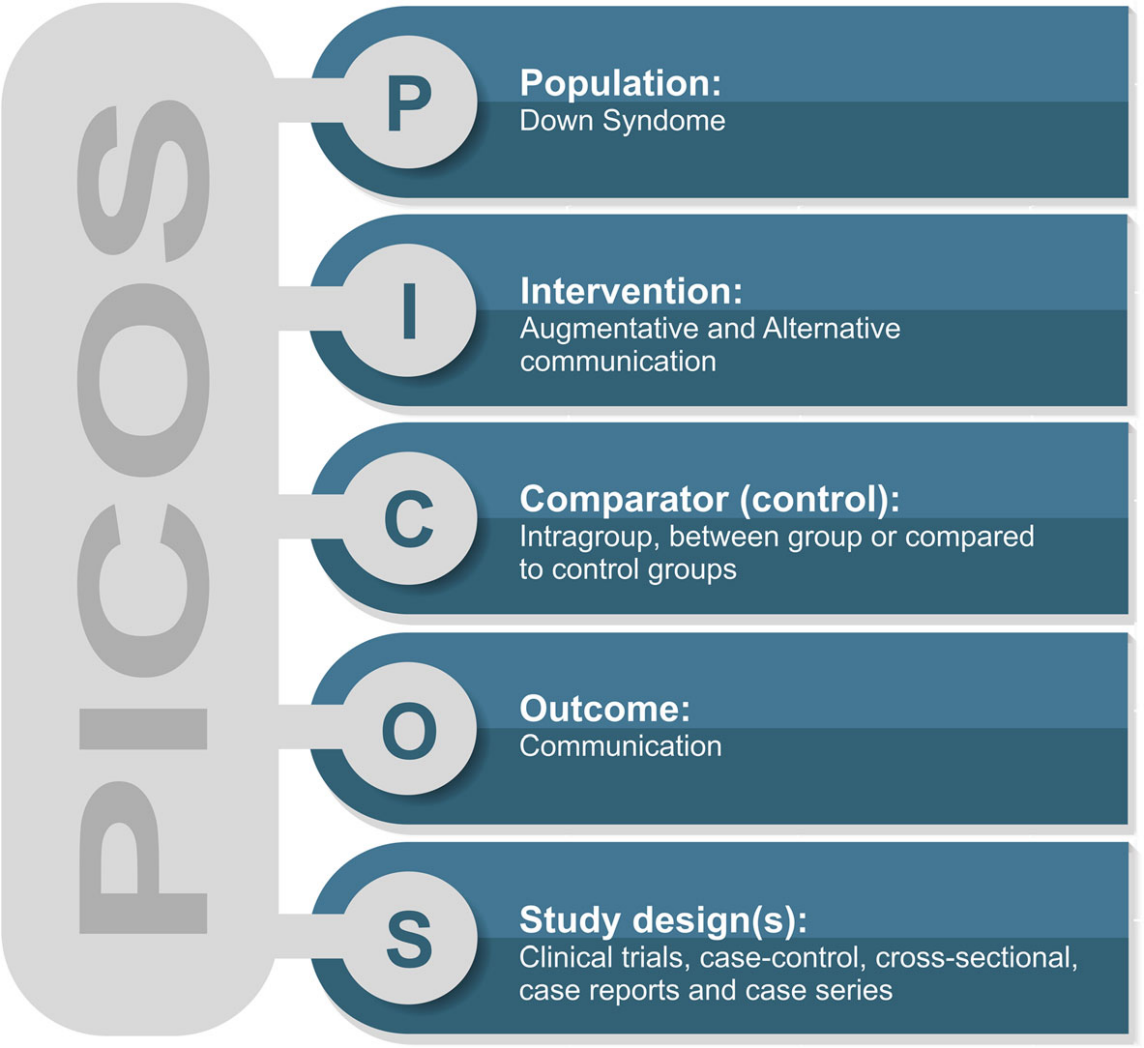
Population Intervention Comparison Outcome Study Design (PICOS) strategy

Finally, reference lists of retrieved studies were hand-searched to identify additional relevant studies. Keywords and a combination of keywords were used to search the electronic databases. We organized the search and selection of studies following the Population Intervention Comparison Outcome Study Design (PICOS) strategy. As performed by Massetti et al. [23], Sampaio and Mancini [24], and Massetti et al. [25], we used the search strategy based on their composition according to the PICOS method to locate and compare different works (Fig. 1). In this model, the search strategy is based on the topics of population (P), intervention (I), control group (C), outcome (O), and study design (S) as well as several searches in the cited databases.

### Selection process

We used three steps to select the articles. The first step involved searching for the articles in the databases and reading the titles and abstracts. The second step involved the exclusion of works using the title or abstract and inclusion criteria analysis. The third and final step was to analyse the full text of the eligible works [26, 27]. After the removal of duplicates, two authors evaluated the titles, abstracts, and inclusion criteria independently.

### Inclusion criteria

Studies published in English were eligible if they met the following criteria: (1) study of children with a diagnosis of DS, and (2) assistive technology and/or AAC analysis in this population. There were no restrictions regarding sample size. To determine the age range limit, we used the chronological limits of the child defined by the Brazilian Child and Adolescent Statute (ECA). This statute defines a typical child as a citizen under 12 years of age [28] and those between 12 and 18 years of age as adolescents. These guidelines also encompass the range limit proposed by the WHO of 9 years, 11 months, and 29 days [29].

In our study, we did not restrict the time of publication or the type of study design; all publications found, except for reviews, were included.

### Exclusion criteria

Articles were excluded if they (1) were not databased (e.g., books, theoretical papers or secondary reviews, reviews, and meta-analyses), (2) were not published in English, or (3) used populations not explicitly identified with a diagnosis of DS.

### Data extraction and study quality

Data from the included studies were extracted using Microsoft Excel 2010. The form included fields to be completed by a reviewer in the following order: (1) study identification (main author’s name, year, and country), (2) study method (type of study, blinding, and secret allocation), (3) target population aspects (age and sex), (4) aspects of the intervention performed (sample size, presence of supervision, frequency, session length, and follow-up), (5) presence of follow-up, (6) loss to follow-up, (7) studied outcomes, and (8) presented results (see Tables 2 and 3).

**Table 2:** Summary of selected studies using instruments to promote communication and socialization among Down’s syndrome children

Author / Year	Country of Origin	Sample	Age / Gender	Instrument Used	Intervention Time	Limitations
Deckers et al., 2017 [61]	The Netherlands	30 children with DS	DS (2 to 7) / 16 F and 14 M	Core vocabulary	15- to 20-min interactions	Three possible limitations of the present study require consideration. First, the sample size is relatively low at just 30 participants. The second limitation is the relatively small language samples collected of 100 words per child. The final limitation is the uneven distribution of words uttered in the three different settings.
Lorah, 2016 [38]	USA	7 students: 5 ASD, 2 DS	ASD (8 to 12) / 1 F and 4 M DS (11 to 12) / 2 M	SGDs (Proloqu2Go ™) and PECS	10 weeks	The first was that baseline data were not collected. The second was the lack of inclusion of a generalization and maintenance measure within the research design. Finally, the inclusion of a standardized preference assessment such as Multiple Stimulus without Replacement would have enhanced the design.
Lanter et al., 2016 [59]	USA	1 child with DS	DS (7 years 8 months) / 1 M	Picture-based strategy	10 continuous sessions	Clinicians and educators should recognize that the methodology used in this case study was more reflective of that which might be conducted in a clinical or educational setting, as opposed to a rigorous research investigation. Although pre-experimental designs exist in the requesting literature using AAC modes of communication, these types of designs fail to provide ‘a convincing demonstration of experimental control’. Pre-experimental designs capture changes in behavior, which explains their frequent use in clinical or educational settings, but do not sufficiently rule out factors beyond the intervention that might explain those changes. Given that the child in this study failed to demonstrate requesting behaviors across multiple baseline sessions, it is unlikely that factors beyond the intervention were responsible for the communication changes observed.
González et al., 2015 [68]	Spain	9 children with DS	DS (9 to 29) / 7 F and 2 M	Interactive digital board prototype (whiteboard)	3 sessions lasting for 1 to 2 h	No limitations disclosed.
Logan et al., 2014 [57]	USA	1 child with DS	DS 1 year and 1 month / 1 F	Modified ride-on car	28 weeks	First, as with any single-subject research design, especially one involving an infant, it is difficult to conclude that changes were a result of the intervention and not simply maturation alone by the subject. It is important to remember that outcome changes are likely due to multiple factors. Second, this study does not meet every criterion of the single-case study research design. Several measures are used that are objective, standardized, reliable, and previously used [18] but are not widely available in a published format with large-scale reference data for comparison. Also, the PEDI was not administered after a reversal/retention phase. Third, it is possible that the extra attention, stimulation, and encouragement received during the intervention phase led to changes in the outcome measures that are unrelated to Natalie’s use of an ROC.
Wilkinson & Mcllvane, 2013 [52]	USA	12 participants with DS and 12 with ASD	DS (7 to 22) ASD (7 to 22) 15 F and 9 M	PCS	2 sessions of 16 trials	Small sample, leading to difficulties in the distribution of subgroups for the instruments used.
Hu et al., 2013 [64]	USA	8 participants with DS and 5 neurotypical children	DS (10–28) TD (10–13) 4 F and 9 M	Input techniques (keyboard, mouse, word prediction, and speech recognition)	2 sessions, taking into account a time of 45 min	The difficulty in accessing many participants, being aware of the severity of the disability, and comparing the performance of the participating groups. In their method, the task was observed by the researchers, and the participants might have felt uncomfortable or distracted. In addition, the time (software usability) was not completely controlled, so some participants had more time to learn and become familiar with the word prediction software. Another point to be considered is that participants are typically less motivated to perform tasks aimed at goals that are not of their choice compared to exploring tasks chosen by participants.
Brady et al., 2013 [37]	USA	93 children: 45 with ASD, 15 with DS, and 33 with other rare diseases	DS (3 to 5) ASD (3 to 5) Other (3 to 5) 20 F and 73 M	PECS, SGDs, and Language Signals System	2 visits, T1 and T2 120 min for each child in T1 and again in T2, 12 months later	Did not record systematically the number of words available for each child through the AAC. Nevertheless, indicative systems such as group size, type of AAC, and language facilitator’s strategies in the classroom were not analyzed. These factors significantly affected the results for acquiring vocabulary, and therefore it was difficult to interpret the longitudinal effects on the instruction variables.
Barker et al., 2013 [36]	USA	83 children: 43 with ASD, 11 with DS, 3 with global development delay, 1 with spina bifida, 4 with CP, 13 with other genetic syndromes, 1 with traumatic brain injury, and 7 with unknown etiology.	DS (3 to 5) ASD (3 to 5) Global development delay (3 to 5) Spina bifida (3 to 5) Cp (3 to 5) Other genetic syndromes (3 to 5) Traumatic brain injury (3 to 5) Unknown etiology (3 to 5) 17 F and 66 M	PECS and SGDs	2 years	Did not consider the cognitive level of children, preventing comparative data reliability.
Van Der Meer et al., 2012 [35]	New Zealand	4 children: 1 with ASD, 1 with MSDD, 1 with DS, and 1 with congenital myotonic dystrophy and autistic-like behaviors.	DS (7) / 1 M ASD (10) / 1 M MSSD (5) / 1 M Congenital myotonic dystrophy (5) / 1 M	SGDs and MAKATON	2 to 4 sessions, 3 to 4 days per week, lasting about 5 min and 10 trials (intervention) and 1 to 6 months’ follow-up	A short intervention time was used, hindering more robust data analysis.
Allsop et al., 2011 [55]	United Kingdom	257 children: 11 CP, 7 varying levels of deafness, 2 global developmental delay, and 1 DS	9 years and 8 months SD = 1.51, age range 4–12 years of age / 134 F and 123 M	Web-based survey (joystick)	3 trials	A limiting factor for a small number of the children with disabilities was language comprehension. The children who participated with genetic disorders such as DS or global learning delay often had an SA in place because of other language comprehension difficulties that occurred in their day-to-day education.
Wilkinson et al., 2008 [51]	USA	26 children: 16 with TD and 10 with DS	DS (11) / 7 F and 3 M TD (3 to 4) / 6 F and 10 M	PCS	Training – 6 sessions; Evaluation – 2 blocks, with 12 experimental stimuli each	Error in recording incorrect stimuli.
Foreman & Crews, 1998 [8]	Australia	19 children with DS	DS (2 to 4) / 8 F and 11 M	MAKATON and COMPIC	4 days	Disclosed no limitations

Legend: *DS* Down’s syndrome, *ASD* autism spectrum disorder, *CP* cerebral palsy, *MSSD* multisystem development disorder, *TD* typical development, *PECS* Picture Exchange Communication System, *SGDs* speech-generating devices, *MAKATON* sign language system, *COMPIC* computer-generated pictographs, *PCS* picture communication symbols, *DD* developmental disability, *AAC* augmentative and alternative communication, *PEDI* Pediatric Evaluation of Disability Inventory, *ROCs* modified ride-on cars, *SA* support assistants, *F* female, *M* male, *SD* standard deviation

**Table 3:** Methods, main outcomes, and methodological score of reviewed studies

Author / Year	Study Design	Methods	Main Outcome	Score PEDro
Deckers et al., 2017 [61]	Cross-sectional study	Spontaneous language samples of 30 Dutch children with DS were collected during three different activities with multiple communication partners (free play with parents, lunch- or snack time at home or at school, and speech therapy sessions). Of these children, 19 used multimodal communication, primarily manual signs and speech. Functional word use in both modalities was transcribed. The 50 most frequently used core words accounted for 67.2% of the total word use; 16 words comprised core vocabulary, based on commonality.	The 50 most frequently used core words accounted for 67.2% of the total word use; 16 words were determined to be core vocabulary based on a commonality criterion. Words in the core vocabulary of young children with DS appear to be similar in syntactic semantic and pragmatic functions to core words identified by research in other populations, although the contribution of content words to the core vocabulary of the children with DS seems higher than in other groups.	5/10
Lorah, 2016 [38]	Experimental	Using an alternating treatment design, teachers and paraprofessionals were instructed to conduct mind training trials using both a PE system and an iPad® Mini with the application Proloqu2Go™ as an SGD, with seven school-aged children with a diagnosis of autism or DS. Following 10 weeks of data collection, the student participants were exposed to a device preference assessment, and teachers completed a social validity questionnaire to assess preference.	The results were consistent with previous research indicating equal acquisition and fidelity of use across both devices, but a general preference for the iPad®-based SGDs.	4/10
Lanter et al., 2016 [59]	Case report	The intervention describes how environmental arrangement and generalized cues were used to promote spontaneous communicative attempts during a reinforcing social-communicative context and explains how prompting and modelling were used to facilitate the performance of effective communication behaviors across multiple requesting opportunities.	The child showed significant increases in his use of functional communication, with collateral gains in speech, as demonstrated by the performance of requests.	2/10
González et al., 2015 [68]	Experimental	The methods and techniques included prototyping, questionnaires (pre–post), thinking out loud, video-recording, and structured observation. In terms of the interaction aspects with the whiteboard, the items evaluated included (a) mouse use, (b) placement of numbers and balls, (c) ball deletion and crossing out, (d) placement of the sign of the operation, and (e) use of sensitive areas established in the worksheet.	The use of the digital board (Divermates – prototype) facilitates the process of interaction and usability, and the attractive design that has been evaluated by the specialists enables it to be adapted to needs related to language, color, font size, use of metaphors, organization, presentation, grouping, and categorization of items. Due to the difficulty in writing, the picture has advantages, since it allows it to be operated with the hand and the difficulties with traditional writing can be forgotten. About subtraction and addition operations, the study population shows that subtraction is more difficult than addition. Participants needed help with fingers and balls (symbolic management) in the numerical counting process (task resolutions).	4/10
Logan et al., 2014 [57]	Case report	Report involving a 13-month-old girl (Natalie) over a 28-week period including 3 evaluation moments: baseline, intervention, and retention. The evaluations were carried out at home with the following schedule: 6 bi-weekly visits for 3 months (baseline), 12 weekly visits for 3 months (intervention), and 4 weekly visits for 1 month (retention). Natalie and her family were recorded in the video during the 28-week study using their ride on car in their home and in the community.	Ride-on car use appears to be feasible, fun, and functional in increasing daily mobility for pediatric populations working toward independent walking.	2/10
Wilkinson & Mcllvane, 2013 [52]	Experimental	Visual perceptual factors such as velocity and precision were evaluated by means of a search task, involving targets that were exposed in different spatial dispositions and internal color in one, with the symbols being grouped by internal color; in the other, the identical symbols had no scheme of agreement.	The visual search was superior in participants with ASD compared to those with DS. In both groups (ASD vs DS), responses were significantly faster when symbols were grouped by color. These results show that the visual and perceptual characteristics of the display may be essential characteristics to be considered during the display construction (device panel).	5/10
Hu et al., 2013 [64]	Experimental	This paper reports an empirical study that investigated the use of three input techniques (keyboard and mouse, word prediction, and speech recognition) by children and young adults with DS and neurotypical children.	Children with TD achieved better performance than participants with DS. The results suggest that some individuals with DS have the skills to enter text at a productive speed and with acceptable accuracy while others are very slow in entering data and the generated text contains a substantial number of errors. The DS group showed a greater variation than the neurotypical group in terms of data entry speed and accuracy.	4/10
Brady et al., 2013 [37]	Longitudinal	These 93 children were assessed at Time 1, followed by 82 of these children after one year being assessed at Time 2. They were exposed to different types of AAC, which were selected by teachers from a list of options, and teachers were asked to record all types of AAC in use for a particular child. Many children have been taught to use multiple forms. Many children added or changed systems throughout this study (therefore, analyses related to specific types of AAC were not possible).	Interventions using symbols by visual image, signs (gestures), and spoken words that may aid in cognitive development and language comprehension (ISF). This hypothetical ISF model reflects positively, since children showed higher levels of ISF, especially those with direct participation of adults at home (domestic environment).	5/10
Barker et al., 2013 [36]	Longitudinal	We developed two surveys (a) to describe children’s use of AAC in preschool classrooms, as well as the use of prompts and question asking, and augmented input by their communication partners; and (b) to describe teachers’ experience, training, and perceived support in providing AAC. We then examined the relationship between children’s experience of AAC, including the use of prompts, question asking, and augmented input by their partners, and the growth of receptive and expressive language for 71 children with developmental disabilities over a two-year period.	It is possible to observe positive effects in the use of PECS and SGDs, since they aid in the production of speech, language expression, and social communication of children with DD. The PECS is the form of AAC most used by schoolchildren. It was observed that more than half of the teachers received vocational training in PECS, while only 25% of the teachers had training in the use of SGDs.	4/10
Van Der Meer et al., 2012 [35]	Longitudinal	We compared speed of acquisition and preference for using SGDs versus manual signing (MAKATON) as AAC options. Four children with DD, aged 5–10 years, were taught to request preferred objects using iPod®-based SGDs and MAKATON. Intervention was introduced in a multiple-probe-across-participants design, and SGD and MAKATON conditions were compared in an alternating-treatment design. A systematic choice-making paradigm was implemented to determine whether the children showed a preference for using SGDs or MAKATON.	All participants showed increased use of SGDs when intervention was introduced, but only three learned under the MAKATON condition. This study shows that individuals with DD often show a preference for different AAC options, and it is important to consider the individual’s preference, as this can be an influential factor in communication skills, communicative function, and ease of acquisition.	3/10
Allsop et al., 2011 [55]	Observational	An interactive web-based survey was developed that stored information within a central database. The survey interface was designed for 4–11-year-olds with and without disabilities. Common accessibility issues were identified using the Web Accessibility Initiative, and then an inclusive design approach was used to improve the usability of the survey interface. The joystick designs were displayed as rotating 3D objects in video clips.	All children could complete the tasks from the survey, although children with disabilities had higher completion times and most required a form of assistance from support assistants and/or sign language interpreters. The use of the web-based survey provided a novel means by which to involve children with and without disabilities in the design of assistive technology devices within a primary school environment.	4/10
Wilkinson et al., 2008 [51]	Experimental	Participants were asked to find a target line drawing among an array of 12. Line drawings represented foods (e.g. grapes, cherries), clothing (e.g. a red shirt, a yellow shirt), or activities (e.g. football, swimming). In one condition, symbols that shared a color were clustered together, creating a subgroup within which to search. In another condition, symbols that shared a color were distributed across the display, allowing each to appear individually.	Grouping symbols of the same color facilitated target location velocity (food, clothing, activities) for all participants in the survey and accuracy for younger children and preschoolers with DS. In the construction of the display design, the internal coloring of the symbols should be considered, especially when dealing with individuals with DS, assisting the visual and perceptual condition.	4/10
Foreman & Crews, 1998 [8]	Experimental	The study used a simple repeated measures technique. All children who participated were encouraged to learn to communicate 12 unique words: three verbal instructions alone, three through the symbol method (COMPIC), three through the signal method (MAKATON), and three through the multimodal method (symbol + sign + verbal). The four treatments (verbal, symbol, signal, and multimodal) were administered successively over four days, with interaction and sequence effects being controlled by a Latin square design.	The multimodal method and signal method resulted in significantly higher scores for all children when compared to the symbol method. However, it can be concluded that the multimodal method of instruction is the most effective way of encouraging children with DS (between the ages of 2 and 4) to communicate the names of objects, since it makes use of three auxiliary tools (verbal, symbol, sign).	4/10

Legend: *DS* Down’s syndrome, *ASD* autism spectrum disorder, *TD* typical development, *PECS* Picture Exchange Communication System, *SGDs* speech-generating devices, *MAKATON* sign language system, *COMPIC* computer-generated pictographs, *DD* developmental disability, *AAC* augmentative and alternative communication, *ISF* intrinsic symbolic factor

After performing the initial literature searches, each study title and abstract was screened for eligibility by RTAB and JYFLA. The full text of all potentially relevant studies was subsequently retrieved and further examined for eligibility. To increase confidence in article selection, all potentially relevant articles were reviewed independently by two researchers (RTAB and JYFLA) [24]. In the case of disagreement between them, a third researcher (TBC) was approached for a solution.

The authors of the study had the following functions: RTAB structured the script and directed the work; RTAB and JYFLA collected the studies and organized the data; TM, TBC and CA structured the method and study analysis; ASBO, CBMM and LCA structured the introduction, discussion, and conclusion; CA and TPCA adapted the work to the English language; RG, TPCA and IMPB helped in the construction of the discussion; and LCA reviewed and generally organized the manuscript. It is important to emphasize that the list of articles from the references was analysed as described by Arab et al. [30]; however, this method did not change the results of the initial search.

### Analyses

The study evaluation was performed using the PEDro scale (see Table 3), one of the most used scales in the rehabilitation area [31]. This scale was developed as a part of the Physiotherapy Evidence Database to evaluate experimental studies and has a total score of 10 points, including internal validity of evaluation criteria and presentation of statistical analysis [31]. These criteria are contained in the Delphi list developed by Verhagen et al. [32] and are used to evaluate items in systematic reviews. According to Maher et al. [33], PEDro score efficiency assesses the reliability of the total score based on judgements of acceptable consensus. We followed the methodological quality proposed by Snider et al. [34] and Massetti et al. [26], which ranked the study-level evidence using the following scoring scale: ‘Excellent’ 9–10, ‘Good’ 6–8, ‘Fair’ 4–5, and ‘Poor’ < 4.

## Results

**Fig. 2:**
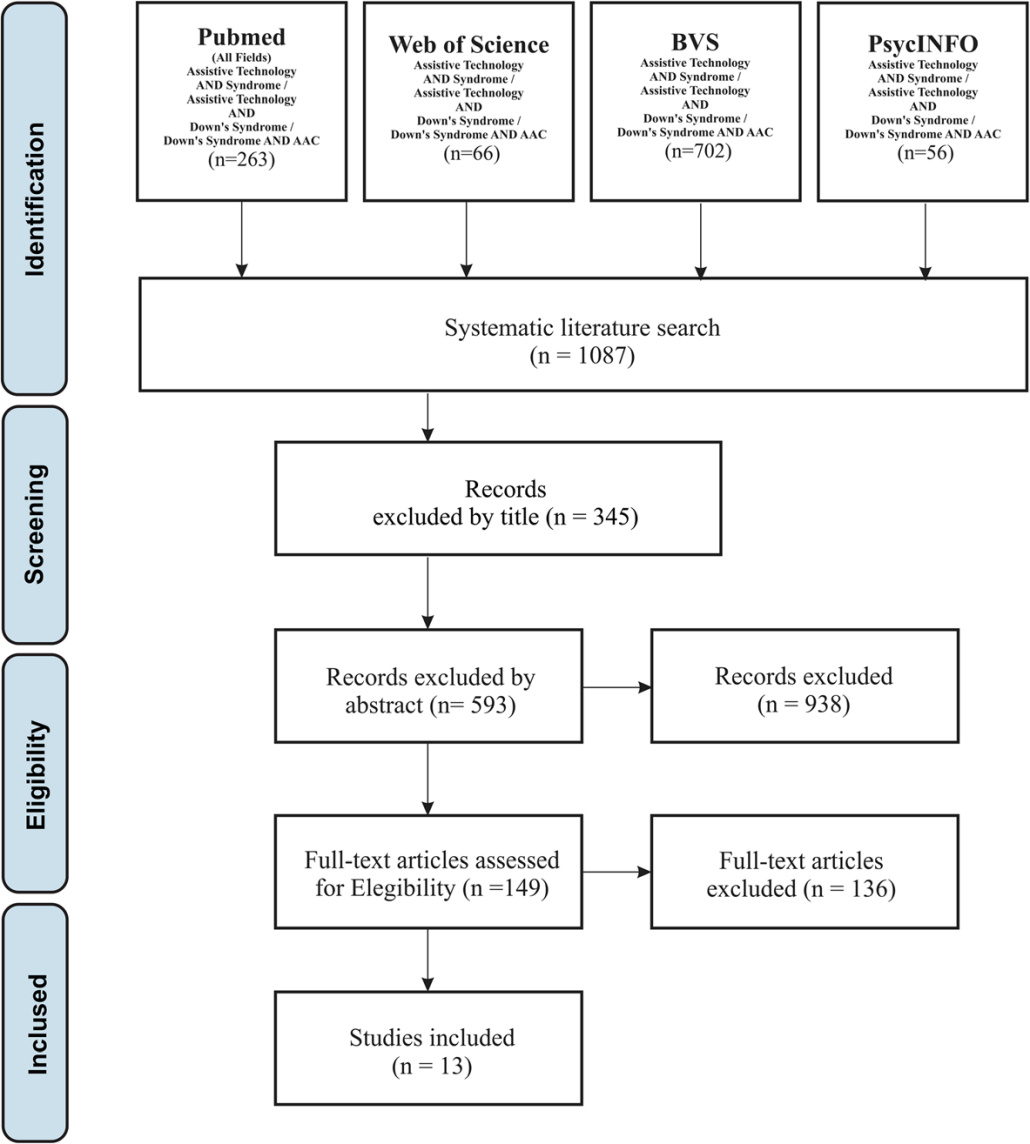
Flow chart of search strategy and selection of the articles. Initialism: AAC: augmentative and alternative communication. Overview of the literature review process. Adapted from Moher et al. (2009)

PubMed, Web of Science, PsycInfo, and BVS database searches resulted in 1087 articles. After filtering articles by reading titles and abstracts, we selected 17 articles for full-text reading. Of these, 13 articles fulfilled the inclusion criteria for this review (Fig. 2) (see Additional file 1, Additional file 2, Additional file 3, and Additional file 4).

## Discussion

The studies used predominantly visual and auditory instruments for communication to provide socialization. We identified twelve instruments used for communication for children with DS (see Table 4) as described below.

**Table 4:** Objectives and characteristics of the instruments used in included studies

Instrument	Instrument Objective	Instrument Features	Number of Studies (Instrument)
SGDs	Improvement of speech (improvement of communication)	SGDs, also known as voice and output communication aids	Lorah, 2016 [38] Barker et al., 2013 [36] Brady et al., 2013 [37] Van Der Meer et al., 2012 [35]
PECS	Broadening of language skills and social communication	Information system through the exchange of image cards (change the image for the item itself) – discrimination of figures, making sentences	Lorah, 2016 [38] Barker et al., 2013 [36] Brady et al., 2013 [37]
MAKATON	Language and communication development (signals domain)	Vocabulary with speech, signs, and/or symbols (interactive vocabulary)	Van Der Meer et al., 2012 [35] Foreman & Crews, 1998 [8]
PCS	Assists the cognitive process (speed and accuracy) through figures and symbols	A pictorial system consisting of designs that mean nouns, pronouns, verbs, and adjectives, or makes use of symbol arrangements (e.g. clothing, shoes, goggles and gloves) used in the “grouped” and “distributed” arrangement with a search focus visual.	Wilkinson & Mcllvane, 2013 [52] Wilkinson et al., 2008
Core vocabulary	Improvement of functional language, seeking to achieve more effective communication	Sets of vocabularies described as basic vocabulary consist of high-frequency words and represent various parts of speech or natural text (i.e. mainly function words such as pronouns, conjunctions, prepositions, auxiliary verbs, determinants, interjections, and adverbs).	Deckers et al., 2017 [61]
Picture-based strategy	Stimulate spontaneous communicative attempts	Three-ring communication folder (~ 7 ″× 5″ × 1 ″) that had three removable pages, each with removable coloured pictures (~ 2 ″× 2″). The first page presents snack items and the second page an immutable series of oral preferred activities reported to promote generalisation. The third page shows a phrase strip indicating the target form (‘I want _____’).	Lanter et al., 2016 [59]
Interactive digital board prototype	Facilitate interaction (social skills) and give personal autonomy	The digital board interface is a subsystem of the Divermates educational system, which provides educational tools with an attractive design.	González et al., 2015 [68]
Modified ride-on car	Improvement of daily mobility, aiding communication and socialization processes, plus the fun factor	Modified touring car	Logan et al., 2014 [57]
Input techniques	Vocabulary analysis, performance, and interaction	Techniques that use computer input devices (mouse, keyboard, word prediction) for evaluating the speed and accuracy of data input	Hu et al., 2013 [64]
Language Signals System	Cognitive development and 2language comprehension	Acceptable approximations of ASL gestures, which use combinations of hand gestures to represent a phrase, word, letter, number, or a combination of these	Brady et al., 2013 [37]
Web-based survey (joystick)	Facilitate socialization and communication	A joystick is an input device, equipped with a lever capable of controlling the movement of a cursor on the screen, and one or more buttons capable of controlling certain actions when pressed. The joystick designs were displayed as rotating 3D objects in video clips.	Allsop et al., 2011 [55]
COMPIC	Language development (symbols domain)	Communication resource consisting of a library of clear and easy-to-understand drawings, called ‘pictograms’, which contain information	Foreman & Crews, 1998 [8]

Legend: *PECS* Picture Exchange Communication System, *SGDs* speech-generating devices, *MAKATON* sign language system, *COMPIC* computer-generated pictographs, *PCS* picture communication symbols, *ALS* American Sign Language

### Primary and secondary outcomes

Given that individuals with Down’s syndrome (DS) have cognitive, language, and socialization deficits and motor delay, it is important to present augmentative and alternative communication (AAC) instruments to this population. The instruments found will be displayed in topics according to the frequency in which they appeared in the results sample. Thus, the instruments will be presented considering the number of studies identified, the number and age of participants with DS and the main improvements brought by the instrument used in DS.

### Speech-generating devices (SGDs)

Four studies [35–38] used *speech-generating devices* (SGDs). These studies included a total of 29 children with DS from 3 to 12 years of age and reported similar results. The efficacy of these instruments in DS was demonstrated by improved communication due to speech improvement, cognition, and socializing. However, the findings from the study by Sigafoos et al. [39] demonstrated that intervention with these instruments only was not sufficient to promote the process of social interaction.

It is interesting to note that SGDs are more frequently used in other populations with developmental disabilities, such as severe apraxia [40] and ASD [41–44], providing communication development through progress in the variables of language, reduction of inappropriate vocalizations, improved social communication, and disruptive behaviour.

SGDs could probably be used more in DS; therefore, teachers should be trained and specialized. According to Barker et al. [36], the Picture Exchange Communication System (PECS) is the most commonly used AAC in DS, and teachers have a higher level of training. In contrast, only 25% of teachers have training in the use of SGDs.

### Picture Exchange Communication System (PECS)

Three studies [36–38] used Picture Exchange Communication System (PECS). These studies included a total of 28 children with DS from 3 to 12 years old. These studies obtained satisfactory results after a follow-up study, and similar results regarding improvements in language skills and social communication were reported.

Converging these findings, the PECS was successfully used to increase interaction among individuals with DS and their peers with a consequent influence on their quality of life [45].

In studies with individuals diagnosed with autism spectrum disorder, the results using the PECS are similar in terms of improving communication and the socialization process [46]. In addition, among preschoolers diagnosed with pervasive developmental disorder not otherwise specified, improvements in spoken communication and an increased number of different words after intervention with the PECS were identified in a six-month follow-up [47].

### Sign language system (MAKATON)

Two studies [8, 35] used a sign language system (MAKATON) with a total of 20 children with DS from 2 to 7 years old. Improvements in language development were noted.

This instrument is being investigated in children as well as adults [48]. Adults with learning disabilities aged 18 to 44 years interacted with researchers through MAKATON signs, assisting in systemic family therapy. Furthermore, the instrument is important in educational environments, i.e., students with difficulties in learning and socialization [49]. For typical individuals and individuals with developmental disabilities, MAKATON can aid in the communication and learning processes, providing educational value and fun [50].

### PCS: Picture communication symbols

Two studies [51, 52] used Picture Communication Symbols (PCS) with a total of 22 people with DS ranging from 7 to 22 years old. This set of symbols is a communication tool that aims to verify the visual perception of the individual with a focus on speed and precision when the symbols are “distributed or grouped” (arrangement) with identical content. PCS is one on of the most widely used commercial AAC symbol sets proposed by Mayer Johnson [52].

Grouping symbols and maintaining their original colour increased the speed for target location (food, clothing, activities) in all the participants, including those with DS and those exhibiting typical development, and precision in children with DS [51]. In the construction of a display design, it is essential to consider visual and perceptual characteristics, especially in individuals with DS [53].

### COMPIC: Computer-generated pictographs

One study [8] used computer**-**generated pictographs (COMPIC) with a total of 19 children with DS from 2 to 4 years old. This study focused on the domain symbols to help identify objects, increase social interaction and language development, and improve their understanding and communication.

In a case study with a 4-year-old child with multiple disabilities, specific COMPIC symbols of his leisure activities (toy cars, blocks, bubbles) were made ​​available. Decision-making and communication development were improved. In addition, an increase in the will to communicate was noted [54].

### Web-based survey (joystick)

One study [55] used a web-based survey (Joystick), with 1 child with DS that was 9 years and 8 months old. It explored a means of retrieving general preferences from children regarding rehabilitation joysticks. The most effective method for designers to use such information remains a challenge (e.g., children’s responses outlining a favourite colour were often different to the colour of their preferred joystick design, so it is unclear how a designer should incorporate this potentially conflicting information).

In addition to children with DS, the joystick has also been used with other populations, such as adults suffering from strokes, resulting in improved functional and cognitive abilities [56].

### Modified ride-on car

One study [57] used a modified ride-on car with 1 child with DS who was 1 year and 1 month old. The method resulted in improved communication and socialization. As noted with any single-subject research design, especially one involving an infant, it is difficult to conclude that changes were a result of the intervention and not simply the subject’s maturation. It is important to remember that outcome changes are likely due to multiple factors, and this study does not meet every criterion of the single-case study research design [57].

A study involving 6 children from 23 to 38 months old with various cognitive and motor deficits revealed that the subjects presented independent mobility and self-initiated interactions with educators and everyday objects [58].

### Picture-based strategy

One study [59] used a picture-based strategy with 1 child with DS who was 7 years and 8 months old. The study established the performance of requests, including multiple opportunities for requesting behaviours in a reinforcing context, environmental arrangement to encourage spontaneous communicative attempts, and the use of prompting and modelling to establish the use of effective forms. This study demonstrates that children with DS can benefit from interventions that use images to facilitate the execution of requests [60].

### Core vocabulary

One study [61] with a total of 30 children with DS from 2 to 7 years old used core vocabulary. The core vocabularies of children in the current study serve several syntactic, semantic, and pragmatic functions. Core vocabulary words contained demonstratives (that, these), verbs (to be, to want), pronouns (my), prepositions (on), and articles (the). Without any known focus on teaching core vocabulary within speech-language therapy, these core words seem to emerge in the spontaneous interactions of the children with DS in the current study either in spoken or signed modalities. This result may not occur in other children with complex communication needs who rely on significant others to add core vocabulary to their AAC devices.

An investigated instrument based on the spoken and signed modalities involving children with typical development [62, 63] had similar results for children with DS.

### Input techniques

One study [64] with a total of 8 people with DS from 10 to 28 years old used input techniques. Computer devices (keyboard, mouse, speech device, and word prediction software) were used for vocabulary analysis, performance measurement (speed and error rate), and assessment of the child’s interaction with the computer [64]. These types of instruments require further analysis given that only a few individuals with DS have the skills/ability to enter a text at a productive speed and with acceptable accuracy. Most people with DS are very slow to enter data, and the generated text typically contains a substantial number of errors.

Children with other disabilities used different devices and showed benefits. Inputs (video cameras, head trackers, and gloves) and outputs (monitors and polarized glasses) attached to computers were also used in deaf children [65]. The focus was on cognitive development, resulting in improved visual and tactile perception. In addition, tetraplegic individuals and patients with neurodegenerative diseases, such as amyotrophic lateral sclerosis, also benefit from emulating the mouse to provide mechanical movements and transform them into electrical signals transmitted via a brain–computer interface [66].

### Language signals system

One study [37] used a language signals system. The study included a total of 15 children with DS from 3 to 5 years old. Positive results were demonstrated with the use of sign language (American Sign Language) to assist with cognitive processes, social interaction, and language development (production of different words) [37]. Harris et al. [67] demonstrated that the use of signs significantly increased communication capacity during the development of children with DS and suggested that early association of signals and active communication by the child may have long-term benefits for development.

### Digital interactive board

One study [68] with a total of 9 people with DS from 9 to 29 years old used a digital interactive board. The study demonstrated significant benefits in terms of socialization, autonomy, and consequently individual self-esteem. González et al. [68] used the Divermates prototype to solve a mathematical task. The results showed that touching the screen improves error correction compared with handwriting, which is especially helpful to individuals with DS given their motor difficulties. In addition to the motivational factor in using computer technology, educational tools with attractive designs enable adaptation according to language needs, colour, font size, organization, grouping, categorization of items, and navigation control. González et al. [69] explain that a customized education system has been created with characteristics that are useful to students with special needs, such as DS. This system assists cognitive and motor skills, playing a key role in the learning process.

### Study limitations

This review employed search terms that exhaustively covered all relevant publications. The review also used structured data extraction and quality appraisal to add to the systematic reviewing methods. Nonetheless, limitations in this review must be acknowledged. For some of the identified studies, weak methodologies and few studies using some presented instruments limited the interpretations and conclusions. We believe that these limitations may be attributed to the fact that research in this area and with this population is still considered recent. The most common design flaws were a) the small number of participants and interventions, b) the lack of a control group, and c) the heterogeneity of the sample. Future research should focus on the gaps that these limitations potentially created in studies with this population. Analysis of the methodological quality of the studies using the PEDro scale reveals that many studies failed to perform randomization and simple blinding, which could make the results more consistent. Moreover, we did not identify studies that were rated as ‘good’ or ‘excellent’, which can be considered a limitation given that most of the included studies were rated as ‘poor’ (3) or ‘fair’ (10).

### Applicability

Devices that help individuals with intellectual and developmental disabilities in schools, associations, or at home daily are important. A lack of support and insufficient training are factors related to the abandonment or limited use of a communication system [70]. Parents, care givers, and the professionals involved must have knowledge about the instrument being used, so they can assist and participate directly in the cognitive and motor development of the individual. Van der Meer et al. [35] noted the importance of taking into account the individual’s preference for different choices of AAC as this factor may influence the communication skills and the acquisition tasks. Foreman and Crews [8] suggest the possibility of combining various methods of communication to enable better development.

### Future perspectives

Follow-up studies and better designed methods are needed so we can follow individuals with DS throughout their development. Furthermore, testing the applicability of various AAC devices is necessary to possibly measure effective perspectives that contribute to communication, socialization, language, and motor control.

## Conclusion

Twelve instruments that significantly aided in the communication and socialization of children with DS were identified from this review. This study highlights that these instruments provide significant results for children with DS not only in terms of their interaction with each other but also their interactions with other people who coexist with this population, thereby improving interpersonal relationships. However, some key factors should be considered in using such technological devices, including preferences, professional and parent training, joint use of the devices, display design, and above all stratification of the cognitive level before any intervention. Future investigations in communication and socialization of children with DS should employ standardized methods.

## Additional files

Additional file 1: Extraction of data – worksheet – Web of Science. (XLSX 54 kb)

Additional file 2: Extraction of data – worksheet – PubMed. (XLSX 75 kb)

Additional file 3: Extraction of data – worksheet – BVS. (XLSX 315 kb)

Additional file 4: Extraction of data – worksheet – PsycInfo. (XLSX 14 kb)

## Abbreviations

AAC: Augmentative and Alternative Communication
ALS: American Sign Language
ASD: Autism Spectrum Disorder
COMPIC: Computer-Generated Pictographs
CP: Cerebral Palsy
DD: Developmental Disability
DS: Down’s Syndrome
F: Female
ISF: Intrinsic Symbolic Factor
M: Male
MAKATON: Sign Language System
MSSD: Multisystem Developmental Disorder
PCS: Picture Communication Symbols
PECS: Picture Exchange Communication System
PEDI: Pediatric Evaluation of Disability Inventory
ROCs: Modified Ride-on Cars
SA: Support Assistants
SD: Standard Deviation
SGDs: Speech-Generating Devices
TD: Typical Development
